# Effects of Al Particle Size on the Impact Energy Release of Al-Rich PTFE/Al Composites under Different Strain Rates

**DOI:** 10.3390/ma14081911

**Published:** 2021-04-11

**Authors:** Liang Mao, Chenyang Wei, Rong Hu, Wanxiang Hu, Puguang Luo, Yuxuan Qi, Chunlan Jiang

**Affiliations:** 1 State Key Laboratory of Explosion Science and Technology, Beijing Institute of Technology, Beijing 100081, China; mliang2015@bit.edu.cn (L.M.); 3120200253@bit.edu.cn (C.W.); 3120205127@bit.edu.cn (R.H.); 1120170351@bit.edu.cn (Y.Q.); 2 Jiangsu Shuguang Guangdian Co., Ltd., Yangzhou 225009, China; 3120180202@bit.edu.cn; 3 Beijing Institute of Space Long March Vehicle, Beijing 100076, China; 3120180188@bit.edu.cn

**Keywords:** PTFE/Al, reactive material, SHPB, explosive loading test, shock-induced reaction

## Abstract

Polytetrafluoroethylene (PTFE)/Al reactive material with different aluminum particle sizes were prepared by molding and sintering, and the effect of aluminum particle size on the impact behavior of PTFE/Al reactive material with a mass ratio of 50:50 was investigated. The results show that aluminum particle size has significant effects on the shock-reduced reaction diffusion, reaction speed, and degree of reaction of the PTFE/Al reactive material. At a moderate strain rate, the reaction delay of PTFE/Al increased, and the reaction duration and degree decreased, with the increase of aluminum particle size. Under the strong impact of explosive loading, aluminum particle size has little effect on the reaction delay, which maintains at about 1.5 μs–2.5 μs, but the reaction durability and degree of reaction of PTFE/Al decrease with increasing aluminum particle size. There is also a strain rate threshold for the shock-induced reaction of PTFE/Al reactive material, which is closely related to aluminum particle size. The shock-induced reaction occurs when the strain rate threshold is exceeded.

## 1. Introduction

As the final damage unit of all kinds of ammunition and missile, warheads have always played an important role in the research of weapon damage technology. Reactive material has become a hot research field because of its unique invasion-detonation joint damage enhancement effect when it is used in a warhead.

Polytetrafluoroethylene (PTFE)/Al composite is a typical reactive material. Different from traditional energetic materials such as high explosives and propellants, PTFE/Al reactive material is usually very insensitive and does not react spontaneously. Under a strong impact load, the Al particles filler and PTFE matrix will react rapidly and violently, and the reaction, which is a detonation-like effect, will release a large amount of heat and energy, and generate gaseous products [1,2,3]. It has been estimated that PTFE/Al reactive material (mass ratio of 26:74) has a calorific value per unit mass of 9.96 MJ/kg, more than twice the calorific value of TNT, and an adiabatic reaction temperature of over 4000 K. It is usually denser than 2.0 g/cm^3^ (1.6 g/cm^3^ for TNT) and has a higher calorific value per unit volume, more than three times that of TNT [4]. Because of the high energy density, good mechanical properties, strong stability, and easy preparation of PTFE/Al, PTFE/Al reactive material has wide range of applications.

Scholars have carried out a series of studies on the properties of reactive material. At present, however, the formulation of reactive material is still a research hotspot—especially research on the influences of different material components on impact reaction, the reaction energy release under explosion load, and so on—constraining the application of reactive material.

As a vital component of PTFE-based reactive material, metallic Al particles determine the thermo-chemical reaction characteristics and mechanical properties to a certain extent [5]. The particle size is an important parameter of metal particles, as different sizes of metal particles may be very different in their physical and chemical properties. In 2000, Styrov [6] found in his experiments that an exothermic chemical reaction occurs when the fluoroplastic penetrators hit the aluminum alloys. Osborne et al. [7] studied the effect of Al particle size on the thermal decomposition of Al/PTFE by Differential Scanning Calorimeter (DSC) and Thermogravimetric Analysis (TG), and the experimental results showed that Al particle size affected the ignition mechanism of materials. The US Navy [8] used a hydrogen gas gun to study the effect of Al particle size on the impact initiation characteristics of PTFE/Al rods. The results show that the threshold and initiation time of PTFE/Al rods change with the particle size. Using a finite element method, Ge Chao et al. [9,10] systematically studied the influence of Al particle size and its distribution on the mechanical properties of Al/PTFE materials from the microscopic point of view. The mechanical properties and reaction of different particle sizes of Al/PTFE materials at medium and low strain rates were studied by Feng Bin et al. [11,12,13]. Wu Jiaxiang et al. [14] have studied the effect of particle size on the quasi-static compression reaction and impact sensitivity of PTFE/Al reactive material. The results showed that with an increase of Al particle size, the toughness of PTFE/Al reactive material increased first and then decreased, while the impact sensitivity gradually decreased. Liu Yuanbin et al. [15] studied the influence of Al particle size on the reaction threshold of Al/PTFE reactive material under SHPB impact. The experimental results showed that the impact reaction threshold of samples increased with the increase of Al particle size.

Shock-induced reaction (SIR) is the key point in engineering applications of PTFE-based reactive material. At present, the research on Al particle size is mainly focused on the mechanical properties and the reaction behaviors under low strain rate load, but little on the effect of Al particle size of PTFE/Al reactive material under high strain rate load. In practical applications, reactive material is often driven by explosives. How to ensure that the PTFE/Al reactive material does not react or reacts less under the strong impact of explosive loading (high strain rate), and reacts violently when impacting the target (medium-high strain rate) is the focus and difficulty of current engineering research.

In this study, Al-rich PTFE/Al reactive material specimens with different Al particle sizes were loaded by split Hopkinson pressure bar (SHPB) test and explosion test. The influence of Al particle size on the impact response of Al-rich PTFE/Al reactive material under different strain rates was investigated by analyzing the test curves and high-speed images. This study is helpful to understand the shock-induced reaction mechanism of PTFE/Al, and may be helpful to the application of PTFE/Al reactive material.

## 2. Experiment

### 2.1. Sample Preparation

**Figure 1:**
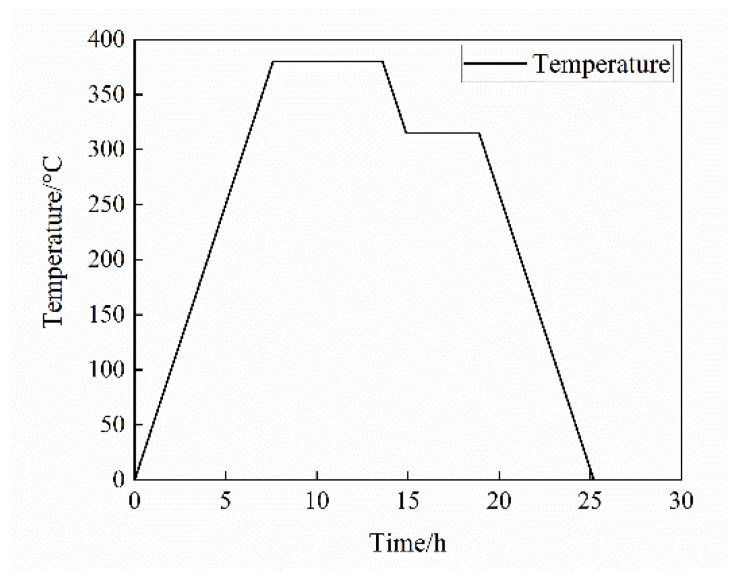
The temperature–time curve of sintering.

In this research, the mass ratio of PTFE and Al is 50:50. High-purity spherical aluminum particles were used as fillers, with a normal particle size distribution and an average particle size of 10 μm, 70 μm, 200 μm, and 50 nm/200 μm. The PTFE/Al reactive materials were prepared by molding and sintering. Firstly, the Al powder and PTFE powder were mixed for 10 h in the medium of a small amount of absolute alcohol, so that the components were mixed evenly. Then the mixed powder was put into the drying oven and dried for 24 h in a vacuum. The mixed powder was molded by uniaxial cold pressing; the molding pressure was 200 MPa, and the holding time was 3 min. Subsequently, the PTFE/Al reactive material cylinders were naturally placed for 24 h to remove the residual stress. Next, the cylinders were sintered in argon atmosphere. The sintering temperature–time curve is shown in Figure 1. Because the PTFE matrix undergoes a phase transition process in the sintering process when the temperature is over 327 °C, the crystalline part of the macromolecular structure changes into an amorphous structure, and the crystalline region disappears completely; when the temperature is lower than this temperature, the recrystallization occurs again, and the crystallinity increases with the cooling rate. In order to ensure the mechanical properties of the sintered specimens, the temperature must be kept at 315 °C for 4 h in the cooling process.

The porosity of the material has an effect on the shock-induced reaction properties [16], so we measured the porosity of the prepared material. The densities and porosities of the four PTFE/Al reactive materials after cold pressing/sintering/machining are shown in Table 1. The PTFE/Al reactive material with different Al particle sizes were less porous, with slightly higher porosity in the PTFE/Al reactive material with mixed 200 μm and 50 nm Al particles.

### 2.2. Dynamic Loading Experiments

#### 2.2.1. Split-Hopkinson Pressure Bar Test

**Figure 2:**
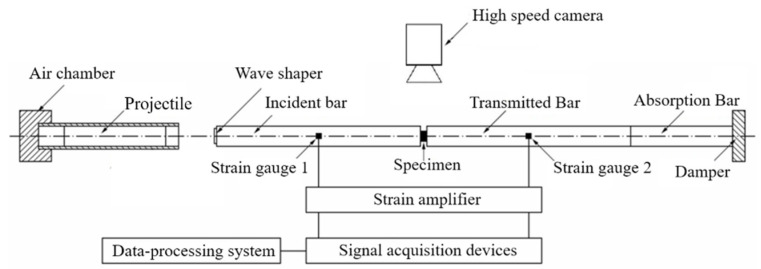
The Split-Hopkinson Pressure Bar Test System.

To explore the influence of Al particle sizes on the impact response of PTFE/Al reactive material under the loading of medium-high strain rates, PTFE/Al reactive materials with different Al particle sizes were loaded by the SHPB test system. The test system is shown in Figure 2. During the test, the high pressure gas drives the projectile to hit the front of the incident bar, and the pulse propagates between the incident bar, the transmission bar, and the specimen.

**Figure 3:**
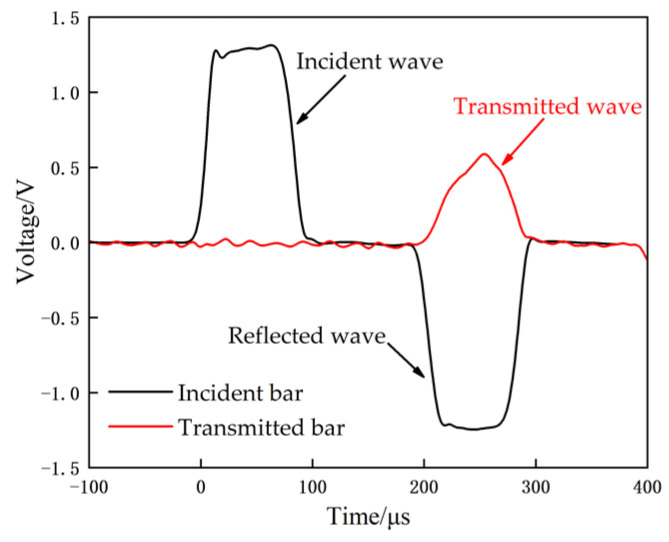
Typical waveforms of incident, transmitted, and reflected waves.

A typical waveform is shown in Figure 3. The incident wave, the reflected wave, and the transmitted wave are measured by strain gauges affixed to the incident bar and the transmitted bar. The strain-stress relationship of the specimen is then derived from the one-dimensional stress-wave theory.

**Figure 4:**
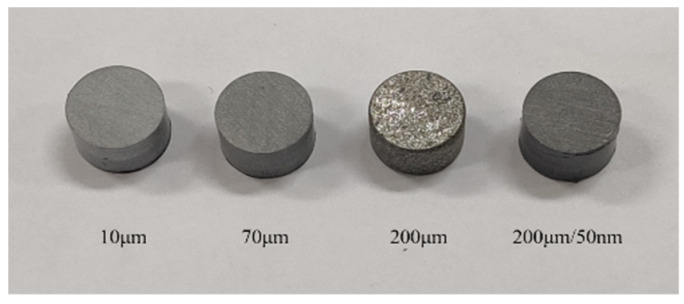
The SHPB test specimens.

Steel bars are used in the SHPB tests. The length of the incident bar and transmission bar is 1200 mm, and the diameter is 16 mm. The length of the bullet is 200 mm. The specimen size is φ8 mm × 5 mm, and the Al particles are 10 μm, 70 μm, 200 μm, and 50 nm/200 μm. The specimens are shown in Figure 4. Compared with traditional metal and alloy, the mechanical strength and impedance of PTFE/Al reactive material are lower. Therefore, waveform shapers are added to the front of the rod to reduce the rise edge of the incident pulse and the signal dispersion.

#### 2.2.2. Explosive Loading Test

**Figure 5:**
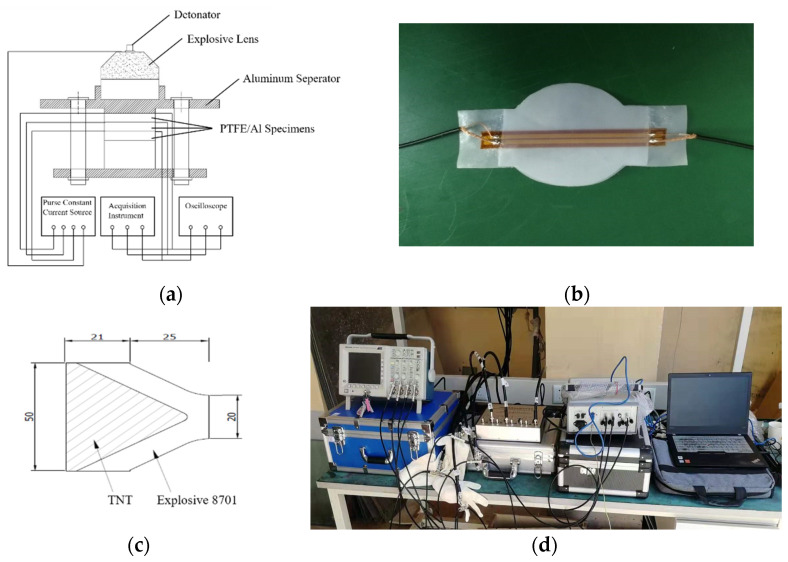
Schematic diagram and object pictures of explosive loading test system. (**a**) Explosive loading test system diagram; (**b**) H-manganin sensor; (**c**) the structure of explosive lens; (**d**) the system layout.

The influence of Al particle size on the impact response of PTFE/Al reactive material under high strain rate was investigated by explosive loading test. The explosive loading test system, as shown in Figure 5, is mainly composed of detonator, explosive lens, electrical probes, aluminum separators, manganin gauges, and PTFE/Al specimens. The explosive lens consists of 39 g of 8701 explosive and 40 g of TNT, the exact dimensions of which can be seen in the Figure 5.

When the detonator detonates, the explosive lens will produce a one-dimensional plane shock wave. At the same time, the electric probe triggers, giving a signal to the pulsed constant current source, and the pulsed constant current source starts to supply power to the gauges. The PTFE/Al specimens are loaded after the attenuation of the 1-D plane shock wave through the aluminum separator. The manganin gauges can be placed at different positions according to the requirements.

The voltage-time signals caused by the shock wave passing through different gauges at different positions are measured and recorded by oscilloscope and acquisition instrument, and the measured voltage signals are converted into pressure values by correlation conversion.

We use low-resistance foil-type H-manganese piezoresistive sensors, encapsulated with PTFE film and vacuum silicone grease. Referring to [17], a 0.1 mm thick PTFE film was used for each sensor. Three manganin gauges are arranged in each group to record the pressure change of each position of the reactive material under the impact loading of the explosive. The first gauge is placed between the aluminum separator and the specimen. A high-speed constant current pulse source, RIGOL DS6000 digital oscilloscope, and TST5205 high-speed collector were used for data acquisition. The acquisition frequency was 50 MHz. Because the loading environment of explosive is very complex, the signal of the gauges will be strongly disturbed with the lapse of time. The effective measuring time of the gauges is about 3–6 μs.

**Figure 6:**
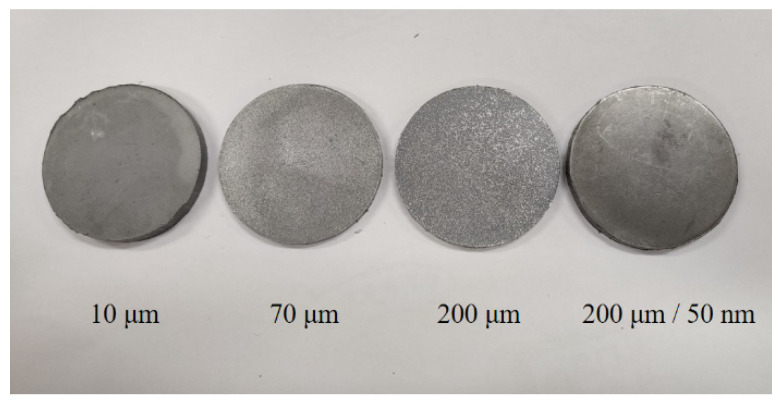
The explosive loading test specimens.

Experiments were carried out on PTFE/Al reactive material with four Al particle sizes: 10 μm, 70 μm, 200 μm, and 200 μm/50 nm. The size of the pieces was φ50 mm × 5 mm and the thickness of the aluminum separator was 13 mm. Figure 6 shows the explosive loading test specimens of PTFE/Al reactive material.

## 3. Results and Analysis

### 3.1. Impact Response of PTFE/Al Reactive Material with Different Al Particle Sizes at Medium-High Strain Rate

#### 3.1.1. Effect of Loading Strain Rate on Impact Response of PTFE/Al Reactive Material

**Figure 7:**
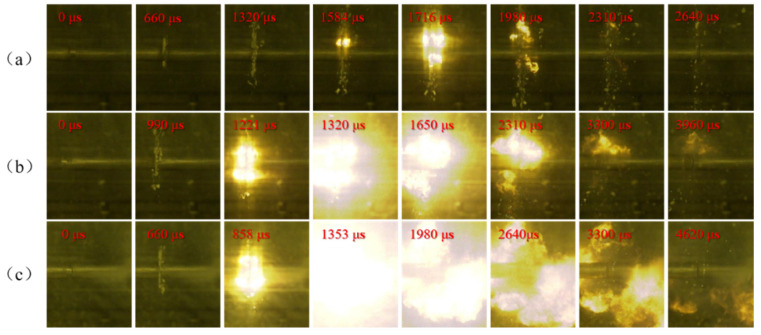
Shock-induced reaction process images of PTFE/Al-10 μm under different strain rates: (**a**) at strain rate 4176 s^−1^; (**b**) at strain rate 4453 s^−1^; and (**c**) at strain rate 5088 s^−1^.

**Figure 8:**
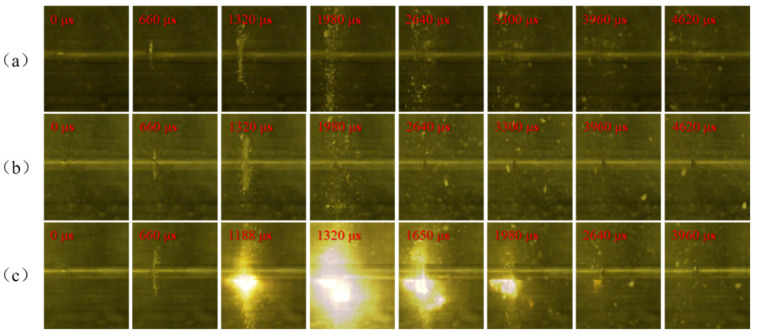
Shock-induced reaction process images of PTFE/Al-70 μm under different strain rates: (**a**) at strain rate 3958 s^−1^; (**b**) at strain rate 4381 s^−1^; and (**c**) at strain rate 5158 s^−1^.

**Figure 9:**
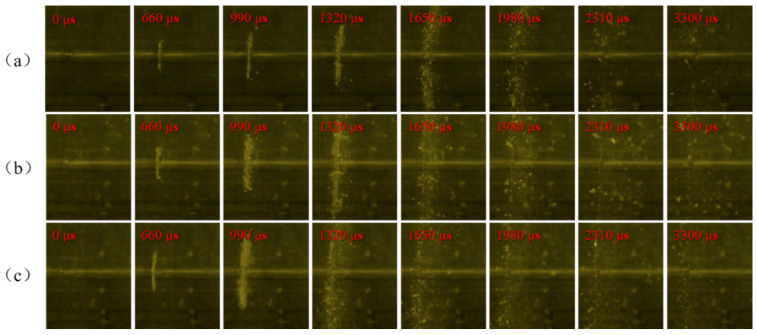
Shock-induced reaction process images of PTFE/Al-200 μm under different strain rates: (**a**) at strain rate 4027 s^−1^; (**b**) at strain rate 4492 s^−1^; and (**c**) at strain rate 5065 s^−1^.

In the experiments, the compression deformation, fragmentation, and ignition process of PTFE/Al specimens with different Al particle sizes under different strain rates were recorded by high-speed camera, and the shooting frame rate was 30,000 frames/second. PTFE/Al reactive material needs certain impact conditions to excite the reaction. Therefore, it is of great importance to study the influence of the loading strain rate on the impact response of PTFE/Al reactive material. Figure 7, Figure 8 and Figure 9 show the shock-induced reaction of PTFE/Al reactive material with different particle sizes under different strain rates. When the particle size of Al is 70 μm and 200 μm, the reaction of PTFE/Al reactive material can be excited only when the strain rate is more than 5000 s^−1^. The reaction propagation of condensed PTFE/Al reactive material is not self-sustained [18]. However, we did photograph the appearance of flame in the SHPB test, so we analyzed the reaction properties of the material by the moment of flame appearance and the duration of the fire. We regard the duration from the moment of initial loading to the moment of appearance of the flame as the reaction delay, and the duration of flame from the appearance to the disappearance as the reaction duration. In the SHPB tests we conducted two tests for each loading strain rate of each material, and the experimental data were reproducible. Table 2 shows the average of the two sets of experimental data.

Shock-induced reaction processes of PTFE/Al-10 μm at different strain rates are shown in Figure 7. The shock-induced reaction is very sensitive to the shock pressure. It can be seen from the figure that with the increase of strain rate, the ignition delay of the specimen decreases gradually, and the reaction duration becomes longer from 957 μs to 3564 μs. There are still some PTFE/Al reactive material fragments around the incident bar when the light disappears in Figure 7a, indicating that a large number of PTFE/Al reactive materials have not reacted. With the increase of strain rate, the PTFE/Al fragments gradually decreased, which means that the mass of reacted PTFE/Al increased along with the increase of the loading strain rate.

There is an obvious strain rate threshold in the reaction of PTFE/Al reactive material, and the strain rate threshold is closely related to Al particle size. When the particle size is 70 μm, the material can only react at a higher strain rate. Compared with PTFE/Al-10 μm, the ignition delay is longer, the fire is weaker, and the reaction duration is shorter. When the particle size of Al is 200 μm, the reaction cannot be observed at any of the three strain rates, which shows that the shock-induced reaction needs higher excitation energy and a higher loading strain rate.

#### 3.1.2. Effect of Al Particle Size on Impact Response of PTFE/Al Reactive Material

The PTFE/Al-10 μm specimen is severely deformed, and a large number of fragments fly away. At 858 μs, the PTFE/Al-10 μm is excited, and with further compression, the flame intensifies and reaches a maximum at 1452 μs, and then disappears at 4422 μs, with a reaction duration of 3564 μs. Compared with the PTFE/Al-10 μm sample, the PTFE/Al-70 μm sample has a relatively weak shock-induced reaction due to the long delay time, weak flame, and short duration.

However, PTFE/Al specimens with a particle size of 200 μm only exhibit mechanical deformation, failure, and scattering. When the impact load increases to the yield stress, the material undergoes plastic deformation, which is macroscopically manifested by the deformation and upsetting of the specimens. At the same time, as the strain increases, the stress continues to increase, and the internal particles come into contact with each other, forming a large number of micro-cracks in the specimen. The micro-cracks keep expanding, and then the whole material is destroyed, the residue splashes out, and the remaining specimen is extruded into a thin sheet by the incident and transmission bars.

The particle size of Al particles has an obvious effect on the reactivity of PTFE/Al reactive material under SHPB impact loading: as the particle size of Al particles increases, the reactivity of PTFE/Al reactive material decreases continuously. This is due to the apparent scale effect of the reactivity of the micron Al particles [19]. The Al particle size has a significant effect on the oxidation process of metals. The smaller the particle size, the larger the specific surface area; the larger its reaction contact area, the more energy is acquires, and the easier it is to ignite.

#### 3.1.3. Impact Response of PTFE/Al Reactive Material with Mixed Al Particles

The impact response of PTFE/Al composites with particle sizes of 50 nm and 200 µm was also investigated to determine whether the impact response of PTFE/Al composites could be modulated by grading of nanometer and micron aluminum. The response characteristics of the two PTFE/Al materials, 200 μm and 50 nm/200 μm, were significantly different.

**Figure 10:**
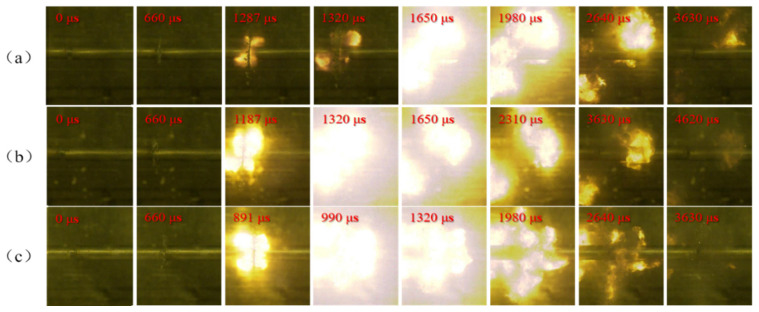
Shock-induced reaction process of PTFE/Al-200 μm/50 nm under different strain rates: (**a**) at strain rate 4001 s^−1^; (**b**) at strain rate 4495 s^−1^; and (**c**) at strain rate 5120 s^−1^.

As can be seen in Figure 10, the reaction of PTFE/Al-200 μm/50 nm clearly burns with a long reaction duration, while the PTFE/Al-200 μm in Figure 9 is only photographed with a large number of material fragments flying out, and no reaction occurs. There are differences in the ignition mechanism between Al nanoparticles and micron Al particles. The ignition reaction of Al nanoparticles can occur at a lower temperature (about 500 °C) than that of micron Al particles, which means that the Al nanoparticles can react with less energy when subjected to impact loading. Therefore, the PTFE/Al-200 μm/50 nm is easier to excite and diffuse after replacing some 200 μm Al particles with 50 nm Al particles.

The shock-induced reaction of PTFE/Al-50 nm/200 μm composites under SHPB loading also exhibited a significant strain rate enhancement. With increasing strain rate, the ignition delay decreases, and the response intensity increases with increasing brightness and area.

### 3.2. Impact Response of PTFE/Al Reactive Material with Different Al Particle Sizes at High Strain Rate

#### 3.2.1. Effect of Al Particle Size on Impact Response of PTFE/Al Reactive Material under Explosive Loading Test

When the plane shock wave generated by the explosive lens reaches the location of the gauges, it first causes a significant pressure jump, and then the pressure decays rapidly [20,21]. For sensors at different locations, the measured peak pressure decreases as the distance the detonation wave travels increases. Over time, the sensors and the circuitry are destroyed by the shock wave, and the signal appears strongly disturbed or interrupted.

**Figure 11:**
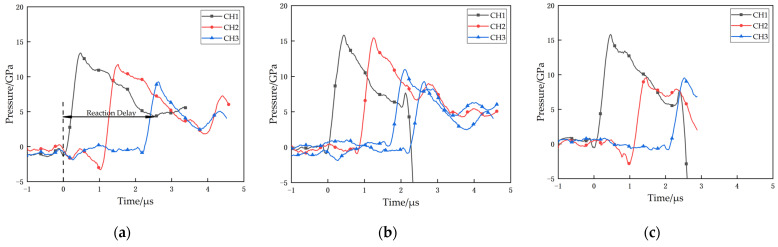
Time-Pressure curve of PTFE/Al of different Al particle sizes under explosive loading test: (**a**) PTFE/Al-10 μm; (**b**) PTFE/Al-70 μm; and (**c**) PTFE/Al-200 μm.

The shock response time-pressure curves for the PTFE/Al reactive material under the explosive loading test are given in Figure 11. For PTFE/Al reactive material, after a short period of pressure decay, a significant pressure curve rebound occurs, as in the curves of channels 1, 2, and 3 in Figure 11a. This is because PTFE/Al reactive material in the sensor area reacts violently after the strong shock wave, and the reaction releases a large amount of energy to make the pressure rise again. However, since the reaction speed of PTFE/Al is lower than the speed of the shock wave, the pressure rebound will occur after the shock wave pressure decays for some time. Referring to the time-pressure data of the three sensors, we take the first pressure jump as the start of the shock wave and the point where the pressure decay curve picks up again as the reaction onset of PTFE/Al reactive material. The explosive loading test data is shown in Table 3.

The time-pressure curves of PTFE/Al reactive material with Al particle sizes of 70 μm and 200 μm under explosive loading are shown in Figure 11b,c, respectively. Similar to the PTFE/Al-10 μm, the same pressure jump and subsequent rapid decay occurs at each sensor location upon arrival of the shock wave. The shock wave pressure decays gradually during the propagation process. In addition, a more pronounced pressure rebound was observed at position 1 around 2 μs after the shock wave reached, which was similar to that of PTFE/Al-10 μm. The difference is that for the PTFE/Al-70 μm, the pressure profile at position 3 did not show a significant rebound after a period of decay, and the pressure was maintained at 8 GPa for about 0.5 μs before decaying again; for the PTFE/Al-200 μm, no significant pressure rebound was measured at position 2 or position 3.

Referring to the experimental results of SHPB test (medium strain rate), it can be seen that as the Al particle size increases, the excitation energy required for PTFE/Al gradually increases, and the reaction speed and the reaction degree gradually decrease. Therefore, when the particle size of Al increases to 70 μm and 200 μm, the PTFE/Al specimen can be excited under higher pressure loading, but the excitation and propagation of the reaction will be more and more difficult, and the reaction velocity and the reaction degree are much lower compared with PTFE/Al-10μm, and only a relatively small amount of reactive material can be excited to participate in the reaction. As a result, the pressure curves at position 3 in Figure 11b and position 2 and position 3 in Figure 11c do not show any obvious pressure rebound, and the pressure of the reactive material reaction could only maintain the pressure for a period of time without decay.

**Figure 12:**
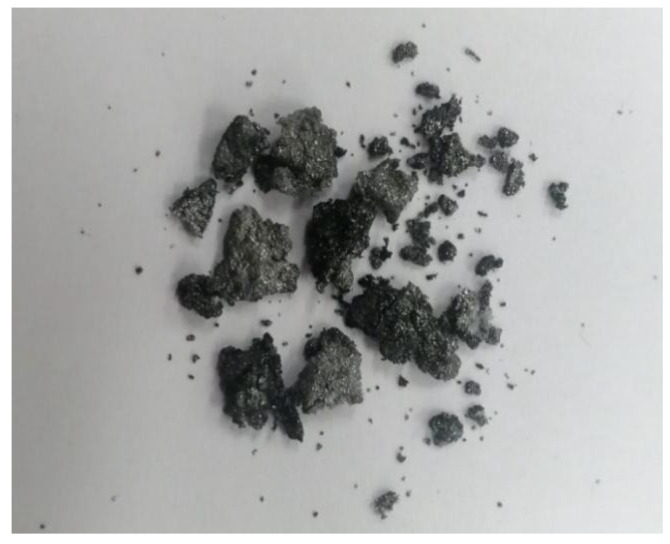
Residue of PTFE/Al-200 μm after explosive loading test.

When the Al particle size increases to 200 μm, the PTFE/Al reactive material suffers from incomplete reaction even under the high strain rate loading of the explosive blast. This incomplete reaction also exists in the explosion loading experiment results of PTFE/Ti [18]. Figure 12 shows the residue collected after the experiment for PTFE/Al-200 μm. It is clear from the figure that the specimens were compressed and sintered together under the high temperature and pressure of the explosive loading, and the specimens showed a certain scorched black appearance, but the presence of Al particles could still be distinguished. No reaction residue was collected in the experiments with Al particle size of 10 μm and 70 μm, which confirms that the reaction rate and reaction degree decrease with increasing Al particle size.

In summary, the Al particle size still has a more obvious effect on the impact response of PTFE/Al reactive material under the strong impact of explosive loading. Compared with the shock-induced reaction experiments at medium strain rate (SHPB), the effect of Al particle size on the reaction delay under high strain rate is smaller, and the reaction delay is basically maintained at about 1.5 μs–2.5 μs. However, the Al particle size has a more obvious effect on the diffusion and reaction degree of the shock-induced reaction of PTFE/Al reactive material. Comparing the maximum pressure rebound values of PTFE/Al with different Al particle sizes, the Al particle sizes from 10 μm to 200 μm are 5.4 GPa, 2.3 GPa, and 2.1 GPa, respectively, indicating that as the Al particle size increases, the ability of PTFE/Al to release a large amount of energy in a short period of time decreases, which means the reaction rate and the degree of reaction decrease.

The pressure records for each material were only obtained from a single test, and the amount of testing was not sufficient. However, the data we collected are in good agreement with the data in the references [22,23,24], so we consider the reliability of the pressure data from this test to be high. However, the material wave velocity of the PTFE/Al-70 um is significantly higher than the remaining groups, which we believe may be due to some defects in the sensor fabrication, the existence of air gaps between the sensor and the material during the assembly process, or the presence of tiny particles on the surface of the specimen, and we will conduct more research subsequently.

#### 3.2.2. Impact Response of PTFE/Al with Mixed Al Particles at High Strain Rate

**Figure 13:**
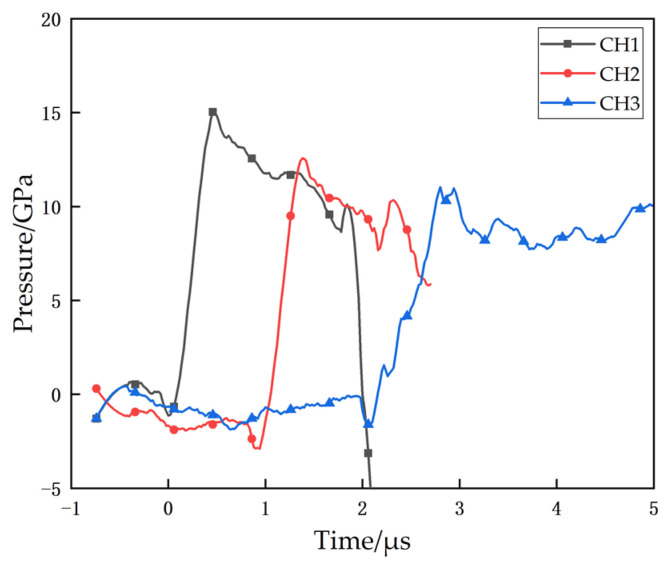
Shock-induced chemical reaction pressure-time curve of PTFE/Al-200 μm/50 nm under explosive loading test.

In the SHPB experiment with medium strain rate, the PTFE/Al reactive material specimens mixed with 50 nm and 200 μm Al particles in a certain ratio showed some improvement in the impact response characteristics in the SHPB experiment regardless. Therefore, this experiment also examined the reaction characteristics of PTFE/Al specimens mixed with 50 nm and 200 μm aluminum particles in a certain ratio under strong impact of explosive loading, and the experimental results are shown in Figure 13.

As can be seen in Figure 13, the addition of Al nanoparticles has a significant effect on the impact response characteristics compared to the PTFE/Al specimen with an Al particle size of 200 μm, with a more pronounced pressure rise at positions 1, 2, and 3. The pressure rebound reached about 3.2 GPa of PTFE/Al-200 μm/50 nm, which is higher than that of PTFE/Al-200 μm at 2.1 GPa. It is worth noting that there is a stable value of about 2 μs at position 3, and the pressure stabilized at about 9 GPa. The aluminum nanoparticles react first, and the reaction of the aluminum nanoparticles further stimulates the reaction of the micron aluminum particles. However, the reaction speed is relatively slow, so the pressure increased by the reaction only appears at position 3, so a stable pressure zone appears at position 3. This phenomenon needs further studies.

**Figure 14:**
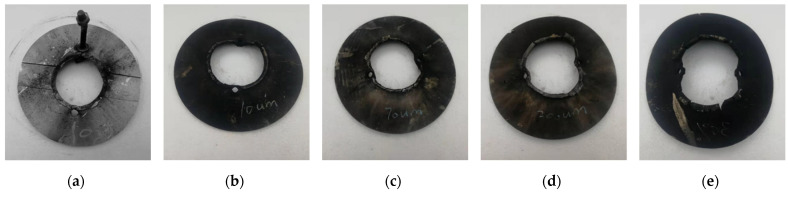
The Al separator surfaces after explosive loading tests: (**a**) surface of aluminum separator of inert materials; (**b**) surface of aluminum separator of PTFE/Al-10 μm; (**c**) surface of aluminum separator of PTFE/Al-70 μm; (**d**) surface of aluminum separator of PTFE/Al-200 μm; and (**e**) surface of aluminum separator of PTFE/Al-200 μm/50 nm.

At the end of the experiments, aluminum separators were collected, and the surfaces in contact with the specimens were compared. Figure 14a shows the surface of aluminum separators of inert material under the explosive loading test. Most of the area remains silvery white in the aluminum separator itself, which indicates that the effect of the explosive on the blackness of the aluminum separator is minimal, and that the blackening of the aluminum separator is mainly caused by the reaction of the adjacent reactive material. Therefore, the reaction degree can be judged to some certain extent from the blackness of the aluminum partition.

It can be seen from Figure 14 that, on the one hand, the degree of blackening of the baffle gradually increases with the decrease of Al particle size, and almost all of the aluminum spacer in the PTFE/Al-10 μm turn black, while only part of aluminum spacer in the PTFE/Al-200 μm turns black. This phenomenon corroborates the above analysis, indicating that the Al particle size still has a relatively obvious effect on the response characteristics of PTFE/Al reactive material under strong impact; on the other hand, the fumigation degree of the aluminum separator with the addition of Al nanoparticles is more obvious than that of the PTFE/Al-200 μm, and the fumigation degree of the aluminum separator demonstrates that the mixture of Al nanoparticles and Al microparticles could modulate and improve the impact response characteristics of PTFE/Al reactive material to a certain extent.

## 4. Conclusions

In this paper, the shock-induced reaction characteristics of PTFE/Al reactive material with different particle sizes were studied under SHPB (medium strain rate) and explosive impact loading (high strain rate); the influence law of Al particle size on the impact response characteristics of PTFE/Al under different loading conditions was analyzed; and the modulating effect of the nano/micron gradation approach on the energy release of PTFE/Al reactive material was also investigated. The main conclusions are as follows:

(1) Under moderate strain rate loading, the reaction delay of PTFE/Al reactive material increases, and the reaction duration and degree of reaction decrease with increasing Al particle size. With the increase of loading strain rate, the reaction duration increases and the reaction delay decreases. The results indicate that reducing the aluminum particle size and increasing the loading strain rate can effectively stimulate the reactions of the PTFE/Al reactive material.
(2) Under the strong impact of explosive loading, the Al particle size has little effect on the reaction delay, which is basically maintained at about 1.5–2.5 μs. However, the particle size of Al has a significant effect on the shock-induced reaction of PTFE/Al. With the increase of Al particle size, the reaction durability and reaction degree of PTFE/Al reactive material decreases.
(3) The shock-induced reaction results of PTFE/Al reactive material under different impact loads show that in the explosion loading experiments, the peak loading pressure is large but the loading time is short, and the reactive material can be excited but the reaction is still incomplete. In SHPB experiments, the loading pressure is lower, but the loading time is longer. When the reaction threshold is reached, the reaction duration is longer and the reaction may be more sufficient.
(4) The PTFE/Al reactive material obtained by mixing 50 nm Al particles with the difficult-to-react 200 µm Al particles showed excellent performance in both moderate and high strain rate impact experiments. The results indicate that the Al nanoparticles/micron particles grading can modify the shock-induced reaction of the PTFE/Al reactive material to some extent.

## Figures and Tables

**Table 1:** Density of the prepared reactive material.

Number	Al Particle Size	Theoretical Density/(g/cm^3^)	Actual Density/(g/cm^3^)	Porosity/(%)
1	10 μm	2.42	2402	0.8
2	70 μm		2398	0.9
3	200 μm		2395	1.0
4	200 μm/50 nm		2385	1.4

**Table 2:** The SHPB test results of PTFE/Al with different Al particle sizes at different strain rates.

Al Particle Sizes	Strain Rate/(s^−1^)	Ignition Delay/(μs)	Reaction Duration/(μs)
10 μm	4176	1584	957
	4453	1221	2739
	5088	858	3564
70 μm	3958	unreacted	-
	4381	unreacted	-
	5158	1155	1782
200 μm	4027	unreacted	-
	4492	unreacted	-
	5065	unreacted	-

**Table 3:** Explosive loading test data.

Al Particle Sizes	Peak Pressure of Shockwave/(GPa)	Ignition Delay/(μs)	Value of Pressure Rebound/(GPa)
10 μm	13.4	2.5	1.3
	11.7	2.9	5.4
	9.3	1.6	2.6
70 μm	15.8	2.0	2.1
	15.4	1.7	2.3
	11.0	1.9	1.8
200 μm	15.8	2.2	2.1
	9.6	1.2	1.1
	9.5	-	-
200 μm/50 nm	15.0	1.8	1.4
	12.6	1.2	3.2
	11.0	1.2	2.4

## Data Availability

Data is contained within the article.
